# Is primary meningococcal arthritis in children more frequent than we expect? Two pediatric case reports revealed by molecular test

**DOI:** 10.1186/s12879-018-3602-y

**Published:** 2018-12-27

**Authors:** S. Ricci, A. Montemaggi, F. Nieddu, D. Serranti, G. Indolfi, M. Moriondo, C. Azzari

**Affiliations:** 1 Immunology Division, Section of Pediatrics, Department of Health Sciences, University of Florence and Meyer Children’s University Hospital, Florence, Italy; 2 Section of Paediatrics, Meyer Children’s University Hospital, Florence, Italy; 3 Section of Pediatrics, Department of Neurofarba, University of Florence and Meyer Children’s University Hospital, Florence, Italy

**Keywords:** Primary meningococcal arthritis (PMA), *N. meningitidis*, Invasive meningococcal disease (IMD), RT-PCR

## Abstract

**Background:**

Primary meningococcal arthritis is a rare infectious disease that occurs in less than 3% of meningococcal infections and is characterized by arthritis without meningitis, fever, rash, or hemodynamic instability Barahona [Case Rep Orthop 4696014:2017 ]. There are no validated clinical criteria that can be used for the diagnosis. We present two pediatric cases of atypical presentation of meningococcal disease revealed by molecular tests.

**Case presentation:**

The clinical presentation of the two children (6- and 9-years-old) was characterized by signs of arthritis. By Real Time Polymerase Chain Reaction (RT-PCR), we identified *N. meningitidis* serogroup Y in the joint fluid in both cases. After specific antimicrobial treatment, the clinical conditions of the two patients quickly improved during hospitalization. *Conclusions.* We believe that the incidence of meningococcal arthritis could be underestimated in those settings where the use of RT-PCR is limited. Clearer data on the incidence of meningococcal disease would help to design specific treatments and the best possible national vaccine strategies [Fiji Sci Rep 23:39784, 2016, J Infect 67:385-90, 2013].

## Background

Primary meningococcal arthritis (PMA) is an uncommon form of meningococcal disease presenting as isolated septic arthritis without any other signs of invasive meningococcal disease (IMD). It is clinically impossible to differentiate PMA from other types of septic arthritis. Synovial fluid culture and molecular tests are pivotal the confirmation of PMA [1–7]. We discuss two PMA cases diagnosed by Real Time Polymerase Chain Reaction (RT-PCR) following the admission in our hospital of two children in January and March 2017, respectively.

### Cases presentation

#### Case 1

A 6-year-old female with Down syndrome presented at our emergency department with a 24-h history of fever, left hip joint pain and limping. She had been previously diagnosed with compensated mitral valve prolapse, reported a past episode of pneumonia and underwent adenotonsillectomy for obstructive apnea 3 years earlier. One month before admission she traveled to Cuba with her family. On admission, she was febrile with left coxofemoral joint pain and movement impairment. Blood tests showed increased white cells count (WCC) with neutrophilia (WCC 23070/mm^3^, N 90%) and a raised C reactive protein (CRP 10 mg/dl, normal < 0.29 mg/dl). A left coxofemoral ultrasound documented a 10 mm intra-articular fluid effusion.

#### Case 2

A 9-year-old male attended the emergency department with a 3-days history of right ankle joint pain non-responding to non-steroidal anti-inflammatory drugs, limited joint movement and apyrexial. The child had been adopted from Hungary the year before and was diagnosed and treated for Toxocariasis at the baseline health screening as an adopted child. There was no recent history of close contact with other children and no history of respiratory or urinary tract infections.

Blood tests showed increased WCC with neutrophilia (WCC 13700/mm3, 70% N), C reactive protein (CRP 11 mg/dl, normal < 0.29 mg/dl), and erythrocyte sedimentation rate (ESR 64 mm/h, normal < 15 mm/h). A right hip X-ray was normal, whereas right hip ultrasound showed an 11-mm intra-articular effusion and swelling of the right synovial capsule. He did not present any other location of arthritis or arthralgia.

#### Case 1 and case 2: Diagnosis, treatment, and outcome

**Fig. 1:**
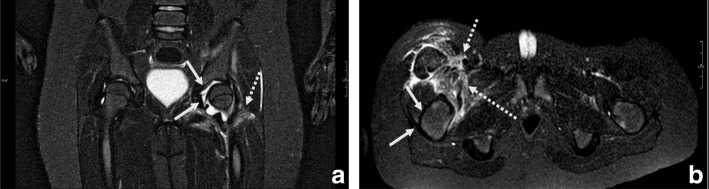
Most significant coxofemoral joint MRI findings performed on the third day after admission in both cases. (**a**). Case I: coxofemoral joint MRI (frontal projection) showed left coxofemoral intra-articular effusion (continuous arrows) with oedematous-flogistic muscular involvement (dotted arrow) but no bone involvement. (**b**). Case II: Coxofemoral joint MRI (axial projection) showed mild right hip joint effusion (continuous arrows) and fasciitis muscular effusion (dotted arrows); no bone lesions were detected

Both patients were admitted to our pediatric ward to be started on empirical antimicrobial treatment with intravenous ceftriaxone and oxacillin for suspected septic arthritis, as recommended in the literature [8]. RT-PCR was performed on the synovial fluid on day 3 of antibiotic therapy: *ctrA* gene and specific primers and probes for A (*sacB* gene) and B, C, W,Y serogroups (*siaD* B, *siaD* C, *siaD* W and Y, respectively) were used for the detection of *N.meningitidis* as we have previously described [9]. The test resulted positive for *N. meningitidis* serogroup Y (NmY). Multilocus sequence typing (MLST) on synovial fluid revealed the presence of the finetype Y P1.5–2, 10–2: F2–13: ST-23 (cc23) in both samples. According to local IMD treatment guidelines, antibiotic therapy was switched to penicillin G and intravenous ceftriaxone was continued while waiting for culture confirmation and MIC (minimal inhibitory concentration) value for penicillin; oxacillin was stopped and prophylaxis for contacts was started [8]. RT-PCR test for *N. meningitidis* in blood and hemoculture (after 24 h of antimicrobial therapy with ceftriaxone and oxacillin) and a synovial fluid culture test (after 48 h of antimicrobial therapy) were negative in both cases*.* No bone involvement was detected with a coxofemoral joint MRI performed in either case (Fig. 1). Our patients’ clinical conditions quickly improved during hospitalization. Intravenous antimicrobial therapy was continued for 15 days and was subsequently switched to oral treatment for a total of 6 weeks. Both patients were asymptomatic at a follow-up arranged 4 months after discharge.

Immunological laboratory screening including serum immunoglobulin, IgG subclasses, antibody response, immunophenotyping, DHR test and complement function assay (CH 50) was performed to investigate any predisposing immunologic deficiency. Down syndrome could be associated with humoral and cellular defects. In case 1, lymphocyte subpopulation analysis showed a mildly reduced CD4+ lymphocytes (24%), with a normal absolute value for age (av: 416 cells/μL). The immunological screening was totally normal in the second case. Neither patient had been previously vaccinated with the quadrivalent meningococcal vaccine which was subsequently offered at follow-up (quadrivalent meningococcal conjugate vaccine and 4CMenB vaccine).

## Discussion and conclusions

*N. meningitidis* (Nm) is a well-known pathogen in meningitis and sepsis, but other end-organ manifestations of IMD such as pneumonia and septic arthritis are also described. Meningococcal epidemiology is unpredictable and not completely understood leading to a high variability in the incidence of IMD [10]. RT-PCR is approximately 3-times more sensitive than culture in identifying *N. meningitidis* from biological samples, therefore meningococcal infections might be underdiagnosed when using culture-based methods alone, especially in the event of an unusual presentation such as arthritis [9, 11–15]. Meningococcal arthritis is a recognized complication of meningococcal infections reported in up to 7.5% of cases in association with IMD. Nevertheless, PMA is an unusual presentation reported in 2.6% of meningococcal infections and has an excellent prognosis [1, 16]. According to Barahona et al. [1], PMA is considered a rare entity, accounting for 1.5–1.8% of all pediatric cases of pyogenic arthritis isolated by synovial fluid culture [17].To our knowledge, less than 50 cases are described in the literature, combining adult and pediatric patients [1–7, 17, 18]. PMA is defined as the presence of septic arthritis without meningeal signs or meningococcaemia and the detection of *N. meningitidis* in synovial fluid and/or blood analysis. The knee is described as the most affected joint followed by the ankle. Approximately 50% of patients were children less than 4 years of age [18]. Despite its unusual presentation it is important not to miss a diagnosis of meningococcal septic arthritis as appropriate treatment and prophylaxis for contacts are required.

When facing uncommon or no specific presentations (e.g., pneumonia, septic arthritis), clinicians are less likely to suspect meningococcal disease and thus to submit clinical specimens for laboratory confirmation than in classic IMD cases (meningitis or sepsis). Moreover, in the context of an unusual presentation is more likely to collect specimens for microbiological identification after having started empirical antimicrobial treatment, reducing the likelihood for the pathogen isolation. This suggests that a proportion of laboratory diagnosis of PMA might be lost and that disease burden PMA might be underestimated. RT-PCR improves the diagnostic rates after having started antibiotic treatment and also allows the characterization of the Nm serogroups, which contributes to a better understanding of the meningococcal disease epidemiology.

A rise in the number of NmY infections has been observed in the last 20 years in several countries [19, 20]. In Italy, despite an overall invariable incidence of IMD of 0.3 cases/100,000 inhabitants since 2007, an increased proportion of NmY has been observed [21]. Similarly, to other European countries, serogroup B and C are responsible for the majority of cases, although the proportion of NmY IMD cases has steadily increased over the years [20], surging from 2% in 2007 to 17% in 2013, in particular among 5- to 14-year-old patients, which is reported to be the most affected group since 2008 [21].

In our laboratory, we routinely use RT-PCR tests on blood and other normally sterile fluids – as previously described [9] – in association with culture to enhance the reliability of the IMD laboratory diagnosis. In the cases we presented, even though arthrocentesis was performed after having started antimicrobial therapy, RT-PCR on synovial fluid was able to detect NmY ST23/clusterA3, whereas the cultures resulted negative.

According to the pubMLST database, among invasive NmY the ST-23/cluster A3 complex (cc23) is the major cluster complex (cc) reported in Italy. The ST-23/cluster A3 complex could account for more than 85% of Italian IMD cases caused by NmY (http://pubmlst.org/neisseria) and five of the total of six cases of meningococcal arthritis caused by NmY are reported (from 2011 to 2018) in Europe were characterized as ST-23/cluster A3 complex (cc23). A limitation of this analysis is that the submission of isolates to the database is voluntary, which might represent a selection bias and underrepresent the real incidence rate.

In conclusion, our report aims to raise awareness among physicians of the atypical presentations of IMD and highlights the diagnostic sensitivity improvements added by the routine use of RT-PCR for IMD, also in the event of unusual presentations, such as PMA. Better quality in laboratory confirmation of IMD is necessary to improve the understanding of the epidemiological features of Nm disease. Currently NmY is not part of the Italian Immunization Program for children under 11-years-old and, based on our limited data we cannot draw conclusions about a dedicated national vaccination strategy. However, we believe that a more accurate surveillance based on cultural and molecular methods would better frame the diffusion of the different meningococcal serogroups allowing a review of the current vaccination strategies. A better estimation of IMD rates, based on active and appropriate surveillance, would likely help in the development of an effective national vaccination program, which remains the best control strategy to prevent invasive meningococcal disease.

## Abbreviations

CRP: C-reactive protein
ESR: Erythrocyte sedimentation rate
IMD: Invasive meningococcal disease
MLST: Multilocus sequence typing
MRI: Magnetic Resonance Imaging
NmY: N. meningitidis serogroup Y
PMA: Primary meningococcal arthritis
RT-PCR: Real Time Polymerase Chain Reaction
WCC: White cell count
